# The Technologically Mediated Motherhood Constellation: Smartphone and AI as Novel Actors in Maternal Psychic Organization

**DOI:** 10.2196/91292

**Published:** 2026-06-23

**Authors:** Luca Cerniglia, Silvia Cimino

**Affiliations:** 1 International Telematic University Uninettuno, UniNettuno University, Corso Vittorio Emanuele II, Rome, Lazio, 00186, Italy, 39 0669201; 2 Faculty of Psychology, Sapienza University of Rome, Rome, Lazio, Italy

**Keywords:** technoference, digital motherhood, artificial intelligence in mental health, relational third, Daniel Stern

## Abstract

The Daniel Stern concept of the motherhood constellation describes a distinctive psychic organization that emerges in the transition to motherhood, structured by 3 relational discourses and 4 thematic concerns: safeguarding infant life and growth, establishing primary relatedness, securing a matrix of support, and reorganizing maternal identity. Formulated in the mid-1990s, this framework preceded the pervasive integration of smartphones, social media, and artificial intelligence (AI) into everyday life. Contemporary mothers now care for infants in environments saturated by online information, connectivity, and AI-mediated support, raising questions about how these factors participate in and reshape the Stern architecture. This theoretical paper argues that smartphones and AI function not as neutral tools but as systemic actors within the motherhood constellation. Drawing on the Stern model, attachment theory, mentalization research, ecological perspectives, sociotechnical theory, and empirical work on technoference, maternal smartphone use, digital parenting, online maternal communities, and AI-based mental health interventions, we conceptualize digital technologies as entities whose affordances co-structure maternal psychic life. Available findings indicate that they simultaneously expand access to information and support, introduce interactional disruptions, and create new, partially algorithmic matrices of support, with effects moderated by patterns of use, maternal reflective functioning, child characteristics, platform design, and socio-structural conditions. We propose a technologically mediated motherhood constellation, in which smartphones and AI enter all 3 discourses (mother–own-mother, mother–self-as-mother, and mother-infant) and all 4 themes. Rather than asking whether technology is “good” or “bad” for motherhood, we outline a spectrum from technology-enhanced to technology-disrupted constellations and derive implications for clinical practice, research, technology design, and social policy.

## Introduction

In *The Motherhood Constellation*, Stern [1] describes a specific configuration of psychic life that comes to the foreground during pregnancy and early motherhood, organizing the mother’s experience around the infant and reshaping her relations to the past, present, and imagined futures. This constellation is structured by concerns about the infant’s life and growth, the establishment of a primary emotional relationship, the securing of a sufficiently reliable matrix of support, and the integration of “mother” into the woman’s broader identity. These thematic concerns are enacted within 3 interlocking discourses: the mother’s dialogue with her own mother and the internalized maternal figure; her ongoing dialogue with herself-as-mother; and the dynamic, moment-to-moment interactional discourse with the infant [1].

Stern’s [2] account is inseparable from his earlier work on infant experience and affect attunement, which conceptualizes infant-caregiver intersubjectivity as being built from microregulations of gaze, vocalization, touch, and rhythm. The motherhood constellation is therefore not an intrapsychic structure operating in isolation, but a relational organization coconstructed with the infant, the family, and the broader sociocultural environment. The matrix of support is not merely instrumental; it enters the mother’s internal world as anticipated and remembered responses that scaffold her sense of being carried while she carries the child [1].

When Stern developed this model in the mid-1990s, everyday communication and support networks were still largely analog. Information about pregnancy and infant care was obtained from health professionals, printed manuals, and intergenerational transmission. Social support was anchored in copresent kin and local communities. Since then, smartphone penetration has reached very high levels in high-income countries, and the early months of motherhood have become densely interwoven with digital media. Qualitative work by Lupton [3] shows that women routinely use websites, apps, blogs, forums, and social media as central resources during pregnancy and early motherhood, describing them as essential for managing anxiety, isolation, and informational uncertainty. Mothers report consulting online sources multiple times per day for questions about sleep, feeding, crying, and health, often before contacting professionals or relatives [4].

Parallel to this informational shift, a rapidly growing body of literature on “technoference” has documented that parental digital technology use can intrude into parent-child interactions in ways that reduce sensitivity and responsiveness, and is associated with child behavior problems [5]. The longitudinal work by McDaniel and Radesky [5] indicates bidirectional associations between parent technology use, parenting stress, and child behavior. Experimental adaptation of the still-face paradigm to smartphone use suggests that even brief episodes of maternal device engagement can elicit infant responses resembling those seen in classic still-face conditions, including increased negative affect and efforts to reengage the caregiver [6].

However, the same period has seen digital media emerge as vital support infrastructures. Online parenting communities provide emotional validation, practical advice, and a sense of belonging, especially for parents who feel isolated [4,7]. Empirically, participation in supportive online communities has been associated with reduced depressive symptoms and increased parenting self-efficacy in some studies [7]. Conversational agents such as Woebot, although not designed specifically for mothers, have shown promising effects in reducing depressive and anxious symptoms in young adults and perinatal populations, suggesting that artificial intelligence (AI)-mediated interaction can play a meaningful role in mental health support [8].

These apparently contradictory findings highlight the inadequacy of simple “screen time” or “digital good versus digital bad” frameworks. If the motherhood constellation is a context-sensitive organization of psychic life, then the introduction of smartphones, social platforms, and AI systems into maternal environments constitutes a structural perturbation. Technologies now enter all 3 discourses of the constellation as quasi-others that speak, respond, withhold, and curate. This study addresses three questions: how smartphones and AI function as actors within Stern’s framework; through what mechanisms they influence maternal-infant coregulation, reflective functioning, and support matrices; and under which conditions they support or compromise the adaptive functions of the motherhood constellation.

We argue that digital technologies neither simply enhance nor straightforwardly disrupt motherhood. Instead, they participate in a technologically mediated motherhood constellation whose emergent properties depend on patterns of use, design features, maternal and infant characteristics, and socio-structural factors. This holistic framing aims to provide more than a mere theoretical update; it serves a crucial pragmatic purpose for both research and clinical practice. By identifying smartphones and AI as active “systemic actors” rather than neutral tools, we offer clinicians a more nuanced vocabulary to assess digital “technoference” not as a simple behavioral habit, but as a structural shift in maternal psychic organization. This perspective allows for the development of targeted interventions that address how digital interactions may either bolster maternal self-efficacy or, conversely, fragment the primary relatedness essential for infant development. Ultimately, the “technologically mediated motherhood constellation” provides a roadmap for navigating the complexities of modern parenting in an era where the “support matrix” is increasingly digital.

## Theoretical Framework

### Core Concepts of the Motherhood Constellation

The Stern motherhood constellation refers to a temporally bounded but developmentally consequential psychic organization that becomes dominant during pregnancy and the first years of the child’s life [1]. Its thematic focus on life and growth—ensuring that the infant survives and thrives—is accompanied by an equally intense concern with establishing a primary relationship that feels emotionally alive and mutually responsive. Simultaneously, the mother must secure a supporting matrix that can carry her in the new role and reorganize her identity so that “mother” becomes integrated rather than dissociated or alien. These concerns permeate perception, fantasy, and action.

The 3 discourses through which these themes are elaborated are central. In the discourse of the mother with her own mother, transgenerational narratives, identifications, and conflicts are revisited and renegotiated. In the discourse of the mother with herself-as-mother, she evaluates, justifies, and sometimes critiques her caregiving self, using cultural and familial ideals as standards. In the discourse of the mother with the infant, she engages in concrete, embodied interaction whose microregulation feeds back into the other 2 discourses [1]. This triadic architecture makes the constellation inherently relational and context-embedded. The Stern framework was derived from a synthesis of longitudinal infant observations and extensive clinical interviews with parents in psychotherapy, rather than controlled laboratory experimentation. This approach allowed him to capture the subjective, phenomenological “internal world” of the parent during this transition. However, since its publication, the theory has been subject to various revisions. Critics have pointed out its traditional focus on the nuclear family, which may not fully represent the diversity of modern parenting structures (eg, single, same-sex, or adoptive parents). Furthermore, while Stern emphasized the “support matrix” as a physical and relational network, contemporary scholars argue that his model can at times overidealize maternal attunement, potentially neglecting the socioeconomic and structural stressors that modern parents face [9].

### Dyadic Regulation, Attachment, and Reflective Functioning

Work on dyadic regulation and attachment clarifies why disruptions in maternal attention may have outsized developmental consequences. The mutual regulation model developed by Tronick and Beeghly [10] conceptualizes infant–caregiver interaction as a process in which both partners attempt to coordinate states, inevitably failing at times but ideally repairing mismatches. Successful repair, rather than perfect synchrony, supports the infant’s sense of efficacy and trust. Ainsworth’s concept of maternal sensitivity—the capacity to perceive, interpret, and respond appropriately to infant signals—emerged as a robust predictor of attachment security in her classic Baltimore studies [11]. The attachment theory by Bowlby [12] cast these patterns in a developmental perspective, emphasizing how early experiences of caregiver availability are internalized as working models of self and other.

Mentalization theory adds a further layer by highlighting the importance of the caregiver’s capacity to see the child as a psychological agent. Fonagy and Target [13] introduced parental reflective functioning as a construct that captures how parents understand and talk about their child’s mind and their own. Later work has shown that higher reflective functioning is associated with more sensitive caregiving and more secure attachment, even under stress [14]. Reflective functioning also shapes how parents use and interpret external advice and support.

Digital technologies intersect with these mechanisms in several ways. Smartphones compete with infant cues for attentional resources, often via deliberately salient notification systems [5,15]. They provide alternative routes for affect regulation, including distraction and remote social contact, which may either support or displace dyadic coregulation. They offer additional “voices”—online communities, influencers, and AI systems—that comment on maternal practices and infant states, thereby entering into the discourse of the mother with herself-as-mother and with her own mother. Understanding maternal-technology relations, therefore, requires situating them within this regulatory and reflective architecture. For instance, AI-driven chatbots, such as Woebot, are increasingly used by women during the perinatal period to monitor mood symptoms and obtain immediate cognitive-behavioral support [8]. These tools effectively act as digital “auxiliary” supports within the maternal psychic space, providing a 24/7 interactive presence that complements the traditional matrix of support, yet also introduces new dynamics of technological mediation in the mother-infant bond.

### Technology as a Systemic Actor: Ecological and Sociotechnical Perspectives

To conceptualize smartphones and AI as actors in the motherhood constellation, we draw on ecological systems theory and actor–network theory. Bronfenbrenner’s [16] ecological model describes development as being shaped by nested systems, ranging from the immediate microsystem of face-to-face interactions to the macrosystem of culture and institutions [17]. Digital platforms complicate this stratification. A smartphone is present in the microsystem—literally on the sofa where the mother and infant interact—yet it is also an exosystemic conduit for work demands, news, and distant relationships, as well as a macrosystemic embodiment of economic and cultural logics.

Actor–network theory, particularly Latour’s [15] work, emphasizes that nonhuman entities such as technologies participate in networks of action through their material properties and designed affordances. Smartphones prompt checking through push notifications, haptic feedback, and interface designs optimized for engagement. Social media platforms algorithmically curate content, promoting certain narratives of “good motherhood” while occluding others. AI systems generate text that appears conversationally responsive and emotionally attuned, even when it is based on statistical patterning rather than genuine understanding. These features shape human behavior without determining it.

In this paper, smartphones and AI are treated as systemic actors whose affordances, constraints, and algorithmic architectures coconstitute the motherhood constellation. They do so by capturing and fragmenting attention, structuring access to information, shaping perceived norms through algorithmic curation, and providing new forms of quasi-relational responsiveness. The question is not whether mothers “use” technology but how these actors participate in and modify the 3 discourses and 4 themes of the Stern framework.

### Technoference as a Mechanism of Disruption

The construct of “technoference,” developed by McDaniel and Radesky [5], captures everyday intrusions of digital devices into family interactions. In their longitudinal study of parents of young children, higher levels of perceived technoference—defined as technology-based interruptions during parent–child activities—were associated with more externalizing and internalizing problems in children, with parenting stress mediating part of this association [5]. This work extends earlier cross-sectional findings linking problematic parent technology use to less responsive parenting and more child behavior problems.

Experimental studies adapt the still-face paradigm to smartphones. Myruski et al [6] asked mothers to interact naturally with their infants, then to engage with their phones for a short period, and then to reengage. Infants exhibited increased negative affect, decreased gaze toward their mothers, and elevated attempts to reestablish interaction during the phone phase, followed by incomplete recovery in the reunion phase [6]. Systematic reviews, including those by Kildare and Middlemiss [18] and by Braune-Krickau et al [19], synthesize accumulating evidence that parental mobile device use is associated with reduced responsiveness, increased child bids for attention, and more conflict around device management [8].

From a motherhood constellation perspective, technoference can be conceptualized as a recurrent perturbation of the discourse between a mother and her infant. The smartphone becomes a third presence in the dyadic field, sometimes silent, sometimes loudly calling the mother away. Its interruptions are not neutral; they are structured by work demands, social expectations of constant availability, and persuasive design features. These microruptures in mutual regulation do not necessarily produce pathology, but they may accumulate in ways that shape an infant’s expectations of caregiver availability and a mother’s self-representations as “distracted” or “failing.”

## Technology as an Actor in the Parenthood Constellation: Empirical Evidence

This section revisits the 4 thematic concerns by Stern—life growth, primary relating, matrix of support, and identity reorganization—in light of empirical work on digital media, smartphones, and AI in maternal contexts.

### Life Growth and Digital Information Seeking

Within the life-growth theme, perhaps the most visible transformation concerns how mothers access and evaluate knowledge about infant health and development. Lupton’s [3] focus-group study with Australian mothers documented that digital media were experienced as central, sometimes indispensable, sources of information during pregnancy and early motherhood. Participants described using websites, apps, blogs, forums, and social media to understand bodily changes, interpret infant behavior, and manage anxiety, often multiple times per day [4]. They valued immediacy, anonymity, and the ability to triangulate among different sources.

An earlier work by Madge and O’Connor [7] on “parenting gone wired” similarly depicted the internet as a vital site of empowerment for new mothers, providing alternative information channels and virtual companionship during isolated periods. Mothers reported that online forums and email lists were often more responsive and less judgmental than offline supports, especially when discussing taboo topics such as regret, sexual difficulties, or ambivalence.

At the same time, research has identified an information-abundance paradox. High-frequency online searching about health has been associated with heightened health anxiety and “cyberchondria,” as described by Starcevic and Berle [20]. In maternal samples, intensive online information seeking has been linked to increased worry, decision paralysis, and diminished confidence when digital sources offer contradictory or alarmist content [21]. Algorithmic curation can amplify such effects by preferentially surfacing engaging, emotionally charged posts, including extreme narratives about risk and harm [22].

Conversational AI complicates the life-growth landscape further. The randomized controlled trial of the Woebot chatbot by Fitzpatrick et al [8] demonstrated that young adults with depressive symptoms who interacted with the AI for 2 weeks showed significantly greater reductions in depression scores than controls, illustrating the feasibility of fully automated conversational cognitive-behavioral therapy. Although not perinatal-specific, such work has inspired perinatal chatbots that offer psychoeducation, symptom monitoring, and problem-solving strategies to pregnant and postpartum women. Early evaluations suggest high acceptability, with mothers valuing 24/7 availability, nonjudgmental tone, and rapid feedback when they fear something is wrong but feel reluctant to “bother” professionals [23].

From a motherhood constellation perspective, these digital and AI systems enter the life-growth discourse as additional “experts” and “consultants.” They coexist with pediatricians, midwives, grandmothers, friends, and manuals, but their temporal characteristics—constant availability, instant response—and discursive style—concise, often prescriptive, sometimes algorithmically optimized for engagement—make them uniquely salient. Some mothers report feeling reassured when digital advice confirms their intuition; others feel increasingly dependent on constant checking, with uncertainty escalating rather than diminishing.

The transgenerational discourse is also affected. Ethnographic and interview studies suggest that mothers use online information both to challenge and to supplement family advice. Some describe the internet as a way to “fact-check” their own mothers and forge more autonomous caregiving identities, while others report that turning first to digital sources has weakened intergenerational conversations, especially when relationships are conflictual or geographically distant [7,24]. Transgenerational knowledge thus becomes one voice among many in a crowded informational field.

### Primary Relating and Interactional Quality

The primary related theme focuses on the establishment of an emotionally attuned, mutually regulated relationship between a mother and her infant. Within this domain, technoference research directly implicates smartphones as actors in the dyadic field. McDaniel and Radesky’s [5] concept of technoference highlights that seemingly minor, everyday technology interruptions during shared family activities are associated with more problematic child behavior. Their longitudinal work showed bidirectional links: parental technology use predicted later child externalizing behavior, and child behavior predicted later increases in technoference, partly via parenting stress [5].

The adaptation of the still-face paradigm to mobile devices by Myruski et al [6] provided an experimental analog. When mothers were instructed to attend to their phones for a brief period during interaction, infants responded with increased negative affect, protests, and attempts to regain attention, and they showed incomplete recovery after the mothers put the phones away [6]. The authors interpret these findings as evidence that device-induced caregiver unavailability may be experienced by infants as a kind of “digital still face.”

Systematic reviews by Kildare and Middlemiss [18], as well as by Braune-Krickau et al [19], converge on the conclusion that parental device use is associated with reduced sensitivity, less verbal and nonverbal engagement, and more conflict around attention and devices [8,19]. A broader review of parental smartphone use and parent-child interactions describes how enhanced connectivity can distract parents from supervising and engaging with children, leading children to engage in risky or disruptive behaviors to recapture attention [8,25].

At the same time, mothers often experience smartphones as essential tools for their own emotional survival. Qualitative studies highlight that many women use phones during feeding, rocking, or contact naps to alleviate boredom, maintain their adult identity, seek support, or distract themselves from intrusive worries [4]. Some report that brief digital “time-outs” make it easier to regulate their own affect and return to the infant with more patience. This suggests that digital tools may support maternal regulation in ways that indirectly benefit the dyad, especially when offline support is sparse.

A more concerning pattern arises when screens are routinely used to regulate infant distress. Radesky et al [26] have described how parents increasingly rely on mobile devices to calm upset children, particularly in public settings or during family stress. Longitudinal work suggests that high reliance on screens for emotion regulation may be associated with slower development of self-regulatory skills and more behavioral difficulties, though causality is difficult to establish [26]. In the terms described by Stern, digital devices may come to occupy positions in the coregulatory system that would otherwise be filled by the caregiver’s face, voice, and body.

A more optimistic strand of research documents how digital media can enhance primary relationships when used intentionally. Video calling enables ongoing contact between infants and distant relatives; infants quickly learn to respond to familiar faces on screen, and such interactions can sustain relationships that might otherwise deteriorate [27]. Apps explicitly designed to support sensitive parenting—for example, by prompting parents to observe and respond to infants’ cues or by suggesting developmentally appropriate play—have shown preliminary promise in increasing parental confidence and observed sensitivity in small trials [23]. In these designs, digital tools are explicitly oriented toward supporting, rather than competing with, mutual regulation.

### Matrix of Support in Digital Contexts

The Stern matrix of support encompasses the interpersonal and institutional resources that sustain the mother in her caregiving role [1]. Digital technologies have become deeply woven into this matrix. The study by Madge and O’Connor [7] on online mothering forums demonstrates that the internet functions as an “intimate public” in which mothers share experiences, seek advice, and normalize distress. Participants describe online communities as accessible, flexible, and less judgmental than offline spaces, particularly for sensitive topics.

Lupton’s research similarly shows that mothers value online groups and social media for the sense of being “in it together,” especially when facing sleep deprivation, loneliness, and contradictory professional advice [3]. These digital communities provide not only informational support but also emotional and identity-related support, with mothers describing experiences of recognition and validation when others articulate similar struggles.

Quantitative research on online peer support for perinatal mental health suggests that participation in moderated, supportive communities can be associated with reduced depressive symptoms and an enhanced sense of competence, though effect sizes are modest and heterogeneity is high [7]. However, passive browsing without interaction appears to be more strongly associated with social comparison and distress [28].

Here, the digital support paradox becomes evident. The same platforms that offer connection also expose mothers to highly curated representations of motherhood. Research on social media and body image has shown that exposure to idealized online images is associated with increased appearance dissatisfaction via social comparison processes [29]. Similar mechanisms likely operate in maternal domains. Studies of “sharenting” and maternal social media use report that mothers often feel pressure to present an image of “good mothering” on platforms like Facebook and Instagram, even when this feels discordant with their lived experience [30,31]. Such pressures can intensify guilt, shame, and self-criticism, especially among mothers who are already vulnerable to depression or anxiety.

### The Algorithmic Matrix of Support: A Structural Reconfiguration

**Figure 1.**
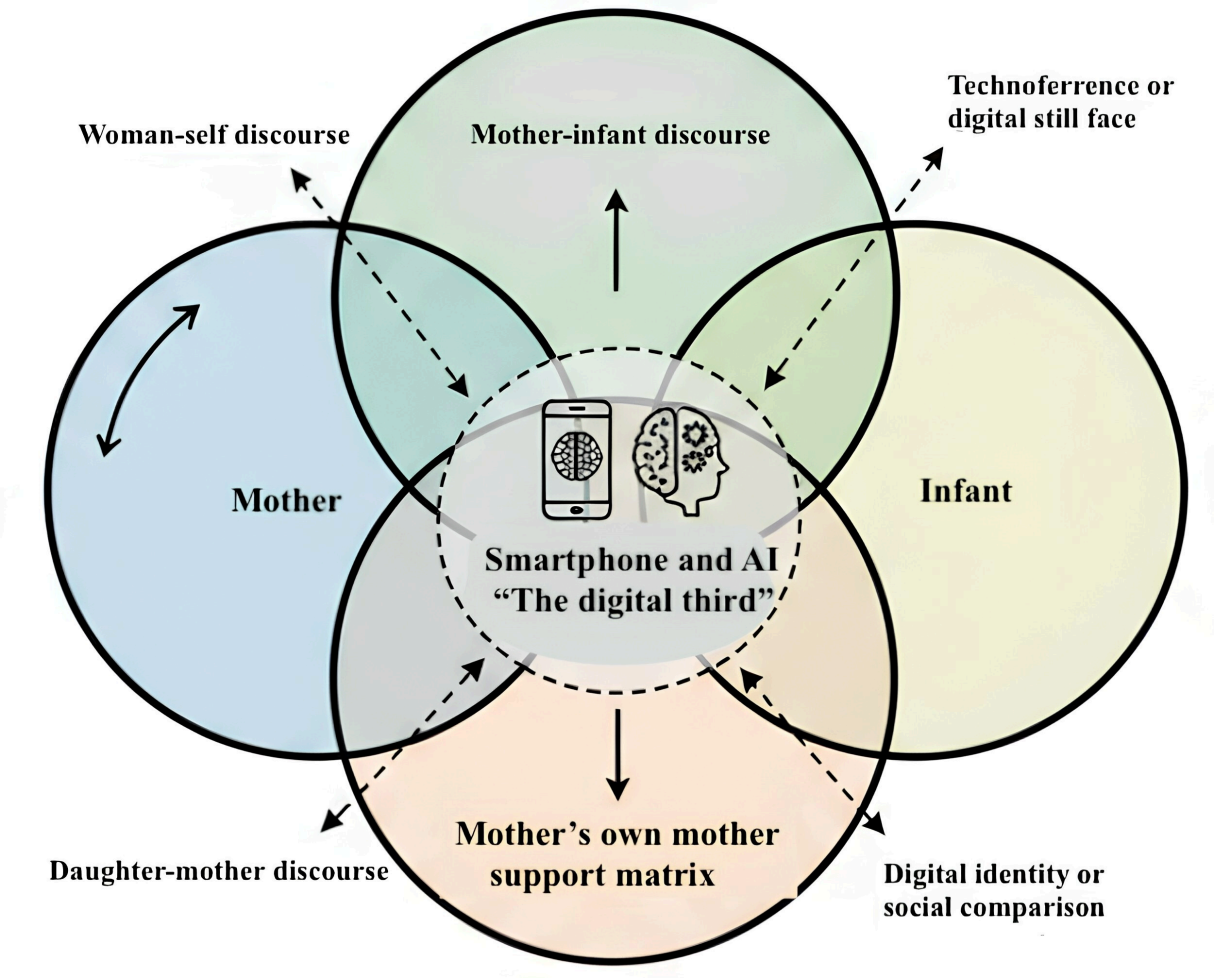
The technologically mediated motherhood constellation framework. AI: artificial intelligence.

In the original framework by Stern, the “matrix of support” is not merely a logistical network but a psychic envelope—akin to Winnicott’s “holding environment” [32]—that shields the mother, allowing her to safely undergo the regression necessary to identify with her infant. We contend that the integration of smartphones and AI induces a structural reconfiguration of this matrix, evolving it from an exclusively human-centric system into a hybrid, algorithmic matrix (Figure 1). In this mediated constellation, technology acts as a digital transformational object that reshapes the maternal experience through several complex mechanisms.

The first mechanism concerns the externalization of reflective functioning, as the use of virtual assistants and data-tracking apps shifts the process of meaning-making from an internal, intersubjective space to an external, objective one (as illustrated in the central node of Figure 1). While Stern described a mother learning to interpret her infant’s cues through intuitive attunement, contemporary mothers often mediate this interpretation through algorithmic outputs. This suggests a risk of dementalization, where numerical data may supersede affective synchrony, transforming the matrix from an emotional container into a technical service provider.

Furthermore, the emergence of the so-called “digital third” causes a disruption of generational linearity, where the support matrix traditionally operated along a mother–own-mother axis, facilitating the transmission of transgenerational wisdom. The contemporary mother frequently experiences tension between the embodied, lived knowledge of her own mother and the optimized knowledge provided by algorithms. This conflict can weaken the daughter-mother discourse, resulting in a support matrix that is highly efficient in information delivery but potentially impoverished in terms of narcissistic support and relational warmth.

Finally, unlike the human matrix, AI is devoid of its own needs and never sleeps, creating an illusion of infinite availability. This hyperavailability induces a distortion in maternal psychic time, potentially bypassing the necessary frustrations and waiting periods that foster resilience. There is a concrete risk that algorithmic support functions as a psychic prosthesis which, while providing immediate anxiety relief, may interfere with the gradual consolidation of an autonomous maternal identity (Figure 1).

AI systems have begun to occupy positions within the support matrix as quasi-relational resources. Mothers using perinatal chatbots report feeling “heard” and “accompanied” during solitary night-time feeds, framing the AI as a safe, always-available listener for worries they might hesitate to share with others [23]. More broadly, work on digital mental health interventions suggests that users can develop attachment-like feelings toward AI “therapists,” experiencing them as supportive companions [33]. In contexts of limited human support, AI may become a primary interlocutor, raising questions about substitution versus supplementation of human relationships.

Digital support is unequally distributed. Research on digital inequalities shows that low-income and marginalized families often have less reliable internet access, lower-quality devices, and fewer digital literacy resources, which limits their ability to benefit from online communities and health information [34]. Within mainstream parenting spaces, mothers of color and other marginalized groups report experiencing racism, classism, and judgment, which leads some to withdraw or create alternative, more inclusive subcommunities [35]. Thus, while digital platforms can expand the matrix of support, they can also reproduce and intensify existing inequities.

### Identity Reorganization in the Age of Sharenting and Metrics

The identity reorganization theme concerns the integration of the maternal role into the self. Digital practices such as sharenting and online self-presentation are now central to this process. Blum-Ross and Livingstone’s [30] analysis of “sharenting” describes how parents share stories and images of their children as part of crafting a digital self that is deeply relational and family-centered. A study by Kumar and Schoenebeck [31] of new mothers on Facebook similarly showed that posting baby photos functions as a “modern day baby book,” a way of enacting and receiving validation for “good mothering.”

These practices can support identity consolidation by providing external recognition and by enabling parents to narrate their experiences. They also create opportunities to challenge restrictive norms by sharing “honest” accounts of difficulty and ambivalence. Yet, they also generate tensions. Some parents experience a gap between the polished maternal persona they feel expected to perform online and the messy, ambivalent reality of their daily lives, leading to feelings of inauthenticity and self-doubt [31]. For others, metrics such as likes, comments, and shares become subtle but powerful validators of maternal adequacy.

Algorithmic curation shapes which maternal identities are most visible. Analyses of influencer culture suggest that high-visibility maternal personas often embody intensive parenting ideals, combining constant child-centeredness with aestheticized domesticity and entrepreneurial self-branding [36]. For parents who do not fit these templates—because of class, race, disability, family structure, or parenting style—digital landscapes can amplify a sense of being “out of place.” Conversely, some parents find in niche online communities, models of motherhood that align more closely with their values, such as queer parenting networks, disability parenting accounts, or antiracist parenting spaces, thereby expanding the repertoire of possible parental selves.

In the terms described by Stern, the discourse of the mother with herself-as-mother is now partially externalized into digital traces that are archived, searchable, and represented via “memories” and recommendation systems. The maternal self becomes a datafied object, visible to others and to future versions of the self. This raises new questions about how the continuity of identity is negotiated when one’s earlier maternal posts resurface years later, sometimes in altered relational contexts.

## Toward a Technologically Mediated Motherhood Constellation

The empirical material reviewed above suggests that the Stern motherhood constellation remains a valuable organizing framework but requires systematic updating for digital contexts. Rather than proposing an entirely new model, we suggest that smartphones and AI are best conceptualized as additional actors within the existing architecture, giving rise to a technologically mediated motherhood constellation.

A central question in this reconfiguration concerns the place of the father and the broader paternal function. In the Stern original formulation, the father appears primarily as part of the supporting matrix rather than as a central organizer of the constellation [1]. By contrast, psychoanalytic and developmental work since Winnicott [32,37] has conceptualized the paternal function as a crucial “third” that interrupts the mother–infant dyad, limits omnipotence, and introduces the child into a shared reality structured by difference, law, and time. Winnicott’s [32,37] account of the transition from subjective omnipotence to objective reality hinges on the infant’s progressive discovery that the mother is not fully under their control, and that there are other relationships and demands that claim her attention, a process that is often mediated by the father or paternal figures. Later authors have elaborated on this notion of thirdness: Ogden’s [38] “analytic third” describes a jointly created psychic field that is neither reducible to 1 partner nor the other; Britton’s [39] work on the parental couple emphasizes that recognizing the parents’ relationship to one another is a developmental achievement that requires relinquishing fantasies of exclusive possession and accepting the limits imposed by an already-existing adult couple. Feldman’s [40] research on triadic synchrony similarly suggests that coordinated patterns of interaction among mother, father, and infant are associated with better socioemotional outcomes, with each caregiver contributing distinct but complementary regulatory inputs. Epidemiological work on paternal perinatal depression underscores that fathers’ psychological functioning is not peripheral; rates of paternal depression in the perinatal period are substantial, and paternal symptoms are correlated with maternal depression and child outcomes [41,42]. Within a technologically mediated constellation, the position of this human third is increasingly shared with, and sometimes displaced by, digital actors. In many families, the everyday triad becomes mother-infant-smartphone, with the device occupying the position of the salient “other” that draws maternal attention away. Crucially, however, the device does not perform the structuring functions of the paternal third. It rarely introduces meaningful, tolerable frustration; instead, it offers a fantasy of limitlessness—endless information, on-demand soothing, perpetual availability—that may collude with omnipotent wishes in both mother and child. Rather than anchoring the dyad in a wider symbolic order, technology frequently presents itself as an extension of individual will and desire. The risk is that digital actors simulate thirdness by inserting themselves into the relational field without providing the kind of containing, law-bearing, reality-introducing presence that human others can offer. When fathers are physically or psychologically present and engaged, they can mediate and modulate digital influences, participating in the coconstruction of rules and boundaries around technology use. When they are absent, overwhelmed, or themselves absorbed in digital environments, the mother-infant dyad may be left to negotiate an asymmetrical relationship with powerful technological actors without the support of a robust human third [40-42]. The technologically mediated motherhood constellation must therefore be conceptualized not only as mother–infant–technology, but as embedded in a wider field that includes, or fails to include, paternal and other third functions.

Within this reconfigured constellation, the discourse between a mother and her own mother is increasingly conducted via digital channels. Messaging apps, video calls, and family group chats enable frequent, sometimes intense contact despite geographic distance. Grandmothers can “be present” at feeding, bathing, or milestones via live video, contributing advice and commentary in real time. At the same time, their authority is subtly relativized as mothers can instantly consult alternative digital sources. The grandmother’s voice becomes one among many, strengthened by digital connection yet challenged by online communities and AI-generated expertise.

The discourse of the mother with herself-as-mother is deeply permeated by algorithmic input. Reflection on one’s adequacy, values, and desires is no longer carried out solely in inner dialogue or in the consulting room, but in interaction with feeds that display other mothers, with notifications that solicit engagement, and with AI systems that provide interpretations and reassurance. The internal critic may be amplified by exposure to idealized images; the internally may be supported by online solidarity and by digital tools that normalize difficulty. For some mothers, conversing with AI systems may function as a form of guided self-reflection; for others, it may externalize and potentially displace their own reflective capacities.

The discourse between mother and infant is altered both directly by technoference and indirectly by the ways digital actors modulate maternal mood, knowledge, and expectations. The smartphone is often physically present in the triad of mother-infant-device; its potential to interrupt is built into its design. Yet, digital tools can also decrease maternal anxiety by providing quick access to trustworthy information or by connecting mothers to supportive communities, thereby freeing attentional and emotional resources for the infant. The same device that disrupts attunement in one context may enable it in another.

These dynamics suggest that the impact of technology on the motherhood constellation is neither uniformly beneficial nor uniformly detrimental. It is better conceptualized as distributed along a spectrum. At one pole lies a technology-enhanced constellation, in which digital actors expand the support matrix, provide reliable information, reduce isolation, and are used in ways that respect and protect the primacy of the mother–infant relationship. In the middle lies a technology-neutral constellation, in which digital tools are present but do not significantly alter core relational processes. At the other pole lies a technology-disrupted constellation, characterized by compulsive use, pervasive technoference, overreliance on digital regulation for both the mother and the infant, and heightened self-criticism driven by social comparison.

Movement along this spectrum is not fixed; mothers may oscillate between more and less adaptive patterns across days, developmental stages, and life events. Crucially, positioning on this spectrum is not solely an individual choice; it is shaped by structural factors such as work demands, availability of offline support, economic pressures, and the business models of platform providers. Recognizing the technologically mediated constellation as a systemic phenomenon rather than an individual failing is essential for ethical responses.

## Clinical and Research Implications

### Clinical Assessment and Intervention

For clinicians working in perinatal mental health and parent-infant psychotherapy, digital technologies have become integral to the ecology of caregiving. Assessment should, therefore, routinely include exploration of maternal technology use. Rather than quantifying “screen time,” the focus should be on patterns, meanings, and contexts: when and why mothers reach for their phones, how they feel before and after digital engagement, what functions different apps serve, and how they experience the impact of technology on their relationship with the infant [43].

Video-based interventions offer a promising route. When mothers and clinicians watch recordings of everyday interactions together, moments of technoference can be observed and reflected upon: a notification that pulls attention away from the infant, a momentary shift in the infant’s affect as gaze is withdrawn, or a tension between the perceived urgency of a message and the infant’s bid for contact. These microevents can serve as starting points for exploring underlying needs, such as the mother’s desire for adult contact, fear of missing work communications, or use of digital distraction to manage anxiety or boredom.

Interventions should avoid moralizing or prescribing blanket digital abstinence. Given that many mothers use digital tools to access support they might otherwise lack, pathologizing technology use risks compounding guilt. Instead, clinicians can collaborate with mothers to develop realistic “micro-boundaries”—for example, device-free feeding routines where possible; silencing nonurgent notifications during certain caregiving windows; or setting aside dedicated times for online support that do not compete with infant cues. Psychoeducation about infants’ needs for contingent responsiveness, the mechanisms of technoference, and the paradoxes of online information can support more intentional use [44].

It is equally important to address the broader support matrix. When digital tools compensate for structural deficits—such as the absence of partner support, lack of paid leave, or limited access to professional care—interventions should validate these realities rather than individualize blame. Clinicians can help mothers identify and mobilize additional offline supports where feasible, and advocate at service and policy levels for conditions that reduce forced overreliance on digital actors [45].

### Research Priorities

Several research priorities emerge from this conceptualization. First, longitudinal studies are needed to examine how early maternal technology patterns relate to longer-term outcomes in attachment, child regulation, and maternal mental health, with attention to bidirectional processes. Most existing work is cross-sectional or short-term experimental; developmental trajectories remain under-specified.

Second, intervention research should move beyond screen-time reduction to test programs aimed at reshaping mothers’ relationships with digital tools. Mentalization-based interventions that include explicit reflection on technology use, design-informed interventions that leverage apps to scaffold attunement rather than distraction, and community-based interventions that build hybrid online-offline support networks are all promising candidates.

Third, AI-specific research is urgently required. We know little about how mothers use general-purpose chatbots for parenting, how they experience AI-mediated support, and what meanings they attribute to these interactions. Mixed-methods studies could explore whether AI support functions as a gateway to human help-seeking or as a substitute for it, and how it influences the discourses of the constellation.

Fourth, moderator research should systematically examine how reflective functioning, attachment history, psychopathology, socioeconomic status, culture, and child temperament shape the impact of digital engagement. Cross-cultural comparative work is essential, as motherhood norms and digital infrastructures differ markedly across societies. Finally, collaboration with human-computer interaction and design researchers is needed to develop and evaluate “benevolent” technologies intentionally designed to support maternal-infant relationships rather than merely to maximize engagement.

### Policy and Design Considerations

Recognizing smartphones and AI as actors in the motherhood constellation has implications for technology design and policy. At the platform level, features that limit intrusive notifications during likely caregiving times, prioritize credible health information, discourage infinite scrolling, and offer clear controls over data collection could meaningfully reduce technoference and anxiety without relying solely on individual self-control [28,46]. Parenting apps and perinatal chatbots should be developed in partnership with clinicians, researchers, and diverse groups of mothers, with explicit attention to safety, equity, cultural relevance, and pathways to human support when needed.

At the policy level, it is crucial to resist framing digital interventions as substitutes for structural support. No chatbot can compensate for the absence of paid parental leave, accessible childcare, or comprehensive perinatal mental health services. Overreliance on technological fixes risks shifting responsibility for coping with structurally generated stress onto individual mothers, exacerbating inequalities. Policies that strengthen the offline matrix of support—economic security, family-friendly work conditions, and equitable health care access—are foundational for any healthy technologically mediated constellation.

Ethical guidelines for AI in health care should be applied rigorously to perinatal applications. These include transparency about AI limitations, clear labeling of AI-generated content, robust data protection, and mechanisms for human oversight and escalation. Given the vulnerability of many mothers during the perinatal period, the commercial exploitation of anxieties through targeted advertising or persuasive design warrants particular scrutiny.

## Limitations and Conclusions

This theoretical synthesis is subject to several limitations. The technological landscape is changing rapidly; platforms and AI capabilities evolve faster than empirical research can track. Some of the specific configurations described here may soon be supplanted by new forms of digital engagement. The empirical literature is heavily skewed toward Western, educated, urban samples; the technologically mediated constellation may look quite different in contexts with different kinship structures, caregiving norms, and digital infrastructures. Many studies rely on self-reported technology use and parenting behavior, which are prone to bias; more work using passive sensing, observational methods, and multiinformant designs is needed. Causal inference remains challenging; children’s behavior influences parental technology use, and maternal mental health both shapes and is shaped by digital engagement.

Moreover, “technology” is not a single entity. Aggregating across platforms, devices, and uses risks obscuring critical differences between, for example, doom-scrolling news during night feeds, participating in a moderated peer-support group, and using a clinically designed app. Future research and theory should differentiate clearly among these modalities and attend to their specific relational affordances.

Despite these limitations, the Stern motherhood constellation remains a powerful lens for understanding early motherhood in digital societies. Conceptualizing smartphones and AI as systemic actors within this constellation clarifies how they participate in the 3 discourses and 4 themes that organize maternal experience. Digital actors expand access to information and support, create new spaces for connection and identity work, and provide tools for emotion regulation. They also fragment attention, introduce new forms of technoference, amplify social comparison, and can displace or distort intergenerational and local sources of knowledge.

The question is not whether mothers should use technology—for most, this is neither realistic nor desirable—but how digital actors can be integrated in ways that support, rather than undermine, life growth, primary relating, the matrix of support, and identity reorganization. This requires interventions at multiple levels: supporting mothers’ reflective and intentional use; designing technologies that respect, rather than exploit, the vulnerabilities of the perinatal period; and addressing structural conditions that currently force many mothers to rely on digital scaffolding in the absence of adequate offline support.

The technologically mediated motherhood constellation is already a reality. The task for clinicians, researchers, designers, and policymakers is to understand its dynamics and shape environments in which digital actors become allies, rather than adversaries, of the maternal and infant needs at its core.

## Abbreviations

AI: artificial intelligence
